# Preserving women’s reproductive autonomy while promoting the rights of people with disabilities?: the case of Heidi Crowter and Maire Lea-Wilson in the light of NIPT debates in England, France and Germany

**DOI:** 10.1136/medethics-2021-107912

**Published:** 2022-03-28

**Authors:** Adeline Perrot, Ruth Horn

**Affiliations:** 1 Nuffield Department of Population Health, Ethox Centre, Wellcome Centre for Ethics and Humanities, University of Oxford, Oxford, UK; 2 Ethics of Medicine, Faculty of Medicine, University of Augsburg, Augsburg, Germany

**Keywords:** Disabilities, Decision Making, Ethics, Genetic Counseling, Prenatal Diagnosis

## Abstract

On July 2021, the UK High Court of Justice heard the Case CO/2066/2020 on the application of Heidi Crowter who lives with Down’s syndrome, and Máire Lea-Wilson whose son Aidan has Down’s syndrome. Crowter and Lea-Wilson, with the support of the disability rights campaign, ‘Don’t Screen Us Out’, have been taking legal action against the Secretary of State for Health and Social Care (the UK Government) for a review of the 1967 Abortion Act: the removal of section 1(1)(d) making termination of pregnancy lawful for ‘severe’ fetal indications detected after 24 weeks' gestation. On 23 September 2021, the High Court dismissed the claim. This action came at a time when non-invasive prenatal testing (NIPT) was introduced into the NHS England Fetal Anomaly Screening Programme for the trisomies 21, 13 and 18. The implementation of NIPT has been heavily criticised, in particular by ‘Don’t Screen Us Out’ campaigners, for increasing fetal selection and discrimination of people living with disabilities. The case of Crowter and Lea-Wilson echoes debates in other European countries such as in France and Germany, where the introduction of NIPT in the public healthcare system has provoked equally vehement public reactions and discussions. The comparison between these three countries allows contextualising the public discourses around NIPT and the ground for termination of pregnancy in relation to different socio-cultural and political contexts. We examine how each country, and particularly England, deals with the conflict between the principles of promoting the rights of people living with disabilities and preserving women’s reproductive autonomy.

## Current controversy

### Introduction

On 6–7 July 2021, Heidi Crowter, who lives with Down’s syndrome, challenged the 1967 Abortion Act (as amended by the Human Fertilisation and Embryology Act 1990) in the UK High Court of Justice. She called for a review of the legal framework, arguing that the current legislation ‘doesn’t respect [her] life’ (BBC news, 6 July 2021) and that ‘people should not be treated differently because of their disability, it is discrimination pure and simple’.^1^ Heidi Crowter has been joined in her legal action against the UK Secretary of State for Health and Social Care by Máire Lea-Wilson whose son Aidan lives with Down’s syndrome. She said: ‘I was 34 weeks pregnant when I discovered Aidan had Down’s syndrome and I was asked if I wanted to terminate the pregnancy in the context of a lot of medically-biased information’ (BBC news, 6 July 2021). Both claimants have been supported by the ‘Don’t Screen Us Out’ campaign, a disability rights campaign that also strictly opposes the implementation of non-invasive prenatal testing (NIPT) by claiming that the test increases the number of terminations of pregnancies (TOPs) for fetuses with Down’s syndrome. The campaign associates NIPT with selective TOP, on the assumption that the availability of NIPT puts undue pressure on women’s choice to undergo testing.

### Public discourses around NIPT as a routine screening test

The legal action against the UK Secretary of State for Health and Social Care came at a time when NIPT is being implemented to improve the existing NHS Fetal Anomaly Screening Programme (FASP) for common trisomies, Down’s syndrome, Edwards’ syndrome and Patau’s syndrome, as part of an evaluative roll out that started on 1 June 2021 in England. It followed the 2016 recommendation of the UK National Screening Committee (UK NSC)^2^, to introduce NIPT in order to reduce the number of women having further invasive diagnostic tests (amniocentesis or chorionic villus sampling (CVS)), which carry a small risk of miscarriage.^3^ Available through the private sector since 2012 in the UK (and in many other countries around the world), NIPT is now publicly offered by NHS England as a second-tier test to pregnant women with a higher probability (1:2 to 1:150) of having a child with one of the trisomies 21, 13 or 18. Compared with the conventional combined first trimester screening (ultrasound and serum markers)^4^, NIPT allows for earlier detection (from 9 or 10 weeks) and higher accuracy in detecting common trisomies with a lower false positive rate.^5^

Recently, also a number of other countries, such as France and Germany, have started to offer NIPT as a publicly funded second-tier test. The public discourses around this test are echoing various socio-cultural and political contexts, and different public health priorities. In France, concerns around NIPT are mainly expressed by the Fondation Jérôme Le Jeune, an association that is close to a conservative catholic milieu. The secular association Trisomie 21 France does not criticise the test itself but stresses the importance of giving women the choice. In the French context, there is a strong focus on the medical expertise and the ‘prevention’ of disabilities at birth.^6^ The introduction of NIPT in the fetal screening policy aims explicitly at increasing the detection rate for trisomy 21^7^ offering NIPT for a lower risk threshold (1:51 to 1:1000) than in England. The argument of improving the detection rate of fetal anomalies by using NIPT is not prominent in Germany and England. These two countries favour the argument of NIPT reducing the number of invasive procedures, and hence of miscarriages. Germany, in particular, is cautious and reluctant to associate NIPT with any form of a population screening programme.^8^ This is to guard against criticism of a possible resurgence of a eugenics programme when making NIPT routinely available.

The introduction of NIPT into the German market in 2012 as well as the parliamentary debate to consider NIPT for public healthcare coverage in 2019 (G-BA) were subject of an extensive public debate and strong criticism by civil society organisations.^9^ In Germany, NIPT will be covered from 2022 by the health insurance in individual cases. No risk threshold is defined for women to access NIPT, as the offer of the test will be based on a case-by-case decision: ‘if, in the course of medical care for pregnant women, the question arises as to whether a fetal trisomy could be present, and the uncertainty is an unbearable burden for the pregnant woman’.^8^

### Issues related to improving information for women and training of health professionals

Despite the advantages of NIPT (easy intervention, accuracy, earlier detection), since 2016 in England, concerns have also been expressed in parliamentary questions from Conservative MPs, campaign groups (‘Don’t Screen Us Out’ and Save Down Syndrome) and a documentary by the British actress, Sally Phillips, ‘A World Without Down’s Syndrome?’.^10^ The concerns expressed focus on the stigmatisation of people living with the condition and the reduction in the number of babies born with Down’s syndrome. These critiques have been raised in particular by the ‘Don’t Screen Us Out’ campaign. To date, however, there is no empirical evidence that the introduction of NIPT has led to increased rates of TOP due to fetal anomalies.^11^ The view of the ‘Don’t Screen Us Out’ campaign differs from that of other disability rights groups such as the Down’s Syndrome Association (DSA), which stresses that it should be up to women to accept or refuse the offer of screening. DSA also emphasises that women should receive up-to-date, accurate and balanced information about people living with Down’s syndrome, and that health professionals should receive regular training regarding how to provide non-directive counselling.^12^

In 2017, a study (Lewis *et al*) has shown that the majority of women who were offered NIPT, made an informed choice (76%) and were judged to have good knowledge (88.8%) about the test and possible results.^13^ These results are close to a Dutch study (TRIDENT) showing that 77.9% of women had made an informed choice for NIPT.^14^ The study conducted by Lewis *et al* raised questions, however, as to whether sufficient time for ‘up-to-date unbiased’ genetic counselling can be provided outside of a controlled research setting.

To address these issues and support informed decision-making, Public Health England (PHE) has developed specific resources for healthcare professionals and women (e.g., an information leaflet,^15^ blog posts,^16^ links to the NHS website^17^ for information and details of support organisations) and training. In addition, the PHE training provided to healthcare professionals who offer NIPT highlights the importance of the language used and the information shared, which must be balanced, accurate and respectful of each individual’s values.^18^ It is also emphasised that positive descriptions of life with the three conditions should be conveyed to pregnant women. This approach differs from France, where there is a stronger focus on the content and amount of the information provided, rather than on the way it is delivered (in a neutral, balanced and non-directive way).^19^ At this point in time, NIPT is introduced in England as an evaluative roll out and the screening pathway may be adapted according to the evaluation.^20^

### Different regulations regarding indications for TOP

Despite the strong emphasis on non-directiveness, in England, activists such as ‘Don’t Screen Us Out’ see the implementation of NIPT into the national screening programme as ‘informally eugenic anti-disabled discrimination’.^21^ Crowter’s and Lea-Wilson’s arguments in the High Court of Justice were that TOP on the ground of disability (Abortion Act 1967) is discriminatory and does not comply with the Equality Act 2010, introduced following the ratification of the UN Convention on the Rights of Persons with Disabilities by the UK government in 2009. In England, medical TOP is lawful provided: ‘(d) that there is a substantial risk that if the child were born, it would suffer from such physical or mental abnormalities as to be seriously handicapped’ (Abortion Act 1967). According to the law, a pregnancy can be terminated at any time up to the moment of birth if a ‘severe disability’ is detected in the fetus. In practice, however, the annual report of the Department of Health and Social Care shows that, for example, in 2020, only 0.1% of TOPs were carried out at 24 weeks and over because of fetal abnormalities.^22^ This raises the question of whether the calls to lower the upper time limit for TOP for fetal abnormalities reflect the reality of clinical practice.

As mentioned above, right-to-life campaigners have regularly been calling to make TOP illegal for ‘severe’ fetal indications after 24 weeks. In July 2013, a committee in UK Parliament held the ‘Disability Abortion Inquiry’ in reference to the Equality Act 2010.^23^ To avoid the discriminatory effect of the Abortion Act, the recommendation to Parliament was to revise the legal framework and reduce the upper time limit for terminations of pregnancy on the grounds of disability as a principle of equality with other fetuses, or to repeal section 1(1)(d) ('severe' fetal indication).

In England, the development of prenatal screening has been criticised as an instrument of fetal selection since 1980s by various advocates such as ‘pro-life’ activists, some feminist groups and some Christian circles. For some of them, these challenges draw on Article 2 of the European Convention on Human Rights—the Right to life—, although the guidelines for the implementation of Article 2^24^ explicitly reject the interpretation that the fetus has an absolute ‘right to life’ and state that ‘its life’ is not considered to have a higher value than the life of the pregnant woman (Article 8: Right to respect for private and family life). This has been also pointed out in the High Court judgment of 23 September 2021 (CO/2066/2020), which rejected the arguments based on Articles 2 and 3, saying that: ‘there was no precedent from the European Court of Human Rights that a foetus has rights under the ECHR’.^25^

The German case offers an interesting point of comparison with the English regulatory framework. The German Criminal Code avoids basing access to TOP on fetal indication (Section 218a of the Criminal Code) and the Embryo Protection Act (1990) aims to protect human life from its beginning. In Germany, the ‘right to life’ (Article 2 of the German Basic Law) extends to ‘every living human being’, including human beings that are yet to be born.^24^

### Facilitating access to antenatal care while promoting inclusion of people with disabilities

In this context of protecting women’s rights, the denunciation of NIPT tends to blur the complexity of individual situations (e.g., socioeconomic, material and time resources of women/couples, different women/couples’ perceptions of disability, past experiences, personal circumstances, family environment) and contexts of variable choices.

In England, while the Nuffield Council on Bioethics report on NIPT^26^ is sensitive to the risks of sending a discriminatory message to families and people living with Down’s syndrome by offering NIPT as part of the screening programme, it also highlights the ethical issues of promoting equitable access to antenatal care and respect for women’s informed choices. The working group supported the introduction of NIPT into the NHS for the common trisomies and highlighted the challenge of demonstrating how society values people with Down’s syndrome, Edwards’ syndrome and Patau’s syndrome by mitigating the negative effects that screening may have. The report emphasises the responsibility to promote inclusion and support people with disabilities in society. This position is similar to that of the German Ethics Council, which stresses that society should do its utmost to enable people with disabilities and their family members to live in society, while taking a clear stance on the fact that ‘stigma and discrimination do not arise as a result of a particular prenatal test, but in the interaction between people’ (Deutscher Ethikrat, 2013).^27^

So far, there is no empirical evidence that the development of prenatal screening tests would have a negative impact on services, care and civil rights of people living with a disability. Nevertheless, the case of Crowter and Lea-Wilson shows that it is important to do more to avoid feelings of discrimination and to acknowledge their rights by offering inclusive policies. Concerns that need to be addressed in relation to the introduction of screening using NIPT relate to improving informed decision-making. It is essential that the principle of reproductive autonomy is not just rhetoric but is implemented in a way that facilitates informed decision-making and mitigates forms of influences on women’s choice. This is particularly important with regard to concerns related to the introduction of this new technology, including the risks of commercialisation and routinisation of NIPT, and medicalisation of pregnancy.^28^ These are only a few of a wider range of concerns around NIPT that need to be investigated thoroughly. Our short contribution focuses on one aspect of the debate and does not reflect the full complexity of the questions and dimensions that are emerging around NIPT.

## Conclusion

In the end, the case of Crowter and Lea-Wilson and activism around prenatal screening highlight the ethical and social challenges facing those offering NIPT as part of routine clinical services in England (France and Germany). They stress the need for pregnant women to receive clear and adequate information about the options available, and that their right ‘not to know’ is respected. To avoid routinisation of screening and protect informed choices, NIPT—just as any other prenatal test—should not be presented as a ‘standard’ test during antenatal care. It is crucial that women have access to appropriate counselling and that they are able to give written informed consent or refuse the offer of NIPT without being subjected to any form of societal, political, economic or medical pressure, and without feeling judged for their beliefs, preferences or values. This legal case underlines the need in England, France, Germany and other countries to reiterate the importance of promoting civil rights, care and inclusion of people with disabilities, while preserving women’s reproductive autonomy. We conclude that both can be defended without having to take sides or neglect either of these two principles.

## Data Availability

There are no data in this work.
